# Combination of Intense Pulsed Light and Topical Eflornithine Therapy Versus Intense Pulsed Light Alone in the Treatment of Idiopathic Facial Hirsutism: A Randomized Controlled Trial

**DOI:** 10.7759/cureus.90414

**Published:** 2025-08-18

**Authors:** Muhammad Usman Nawaz, Muhammad Bilal Baig, Syed Muhammad Shahid, Muhammad Hassan Nawaz, Sara Inayat, Mahrukh Dawood

**Affiliations:** 1 Internal Medicine, Midland Metropolitan University Hospital, Smethwick, GBR; 2 Dermatology, Sandeman Provincial Hospital, Quetta, PAK; 3 Internal Medicine, Heartlands Hospital, University Hospital Birmingham NHS Foundation Trust, Birmingham, GBR; 4 Internal Medicine, Jinnah Hospital/ Allama Iqbal Medical College, Lahore, PAK; 5 Dermatology, Bolan Medical College/Sandeman Provincial Hospital Quetta, Quetta, PAK; 6 Internal Medicine, Pakistan Institute of Medical Sciences, Islamabad, PAK

**Keywords:** facial hair reduction, hirsutism treatment, hormone-independent hirsutism, idiopathic facial hirsutism, intense pulsed light, ipl monotherapy, laser hair removal, topical eflornithine

## Abstract

Background: Idiopathic facial hirsutism (IFH) exerts a measurable negative impact on psychosocial well-being, even in the absence of identifiable endocrine dysfunction. Although light-based epilation technologies offer durable hair reduction, treatment-resistant regrowth remains a clinical challenge. Adjunctive topical therapies that modulate follicular activity are under investigation to potentially enhance and prolong the efficacy of such interventions.

Objective: The objective of this study is to compare the efficacy and tolerability of six-month sessions of intense pulsed light (IPL) combined with a topical agent versus the same IPL protocol alone in Pakistani women with IFH.

Methods: In this open-label randomised controlled trial, 152 women aged 18-70 years were randomised 1:1 to combination therapy (n = 76) or IPL alone (n = 76). The primary outcome was the proportion of participants achieving ≥ 1‑grade reduction on the modified Ferriman-Gallwey (mFG) scale at week 24. Secondary outcomes included mean percentage terminal-hair reduction and patient satisfaction.

Results: Baseline characteristics were comparable. At week 24, 89.5 % of the combination group met the primary endpoint compared with 69.7 % receiving IPL alone (absolute risk difference 19.8 %, p = 0.003). Mean terminal-hair reduction was 90 % versus 59 % (p < 0.001). Treatment-related adverse events were limited to transient erythema and xerosis without between-group differences.

Conclusion: Adding a topical agent to IPL significantly improves clinical and patient-reported outcomes in IFH without increasing toxicity. The regimen may offer a practical, hormone-free first-line strategy for South-Asian women seeking rapid, durable cosmetic benefits.

## Introduction

Hirsutism, the excessive growth of terminal hair in women in a male pattern distribution, affects between 5 % and 10 % of reproductive‑aged women worldwide and represents one of the most common reasons for dermatological consultation [1]. In South Asian populations, reported prevalence rates of hirsutism vary between 10% and 17%, with idiopathic cases accounting for approximately one-third to one-half of all presentations in tertiary dermatology clinics [2,3]. Such figures may underestimate the true burden, as sociocultural factors and limited access to specialist care often delay medical consultation, particularly in rural settings.

Idiopathic facial hirsutism (IFH) is diagnosed when clinical hirsutism occurs in women with regular ovulatory cycles and normal biochemical androgen levels, implying heightened pilosebaceous sensitivity rather than systemic hyper‑androgenaemia [4]. Psychosocial sequelae include reduced self‑esteem, depression and social withdrawal, leading many sufferers to seek repeated, often costly, cosmetic procedures [5].

Among energy‑based hair‑removal devices, intense pulse light (IPL) has gained popularity because of its broad wavelength spectrum (500-1200 nm), large spot size and capability of treating wide skin areas rapidly [6]. By exploiting the principle of selective photothermolysis, IPL targets chromophore‑rich follicular melanin, inducing thermal coagulation of the hair bulb and bulge stem cells. Multiple randomised trials demonstrate 60-75 % hair‑reduction after 4-8 sessions, yet complete clearance is uncommon and relapse rates climb beyond six months [7,8]. Strategies that prolong follicular telogen or delay anagen re‑entry may therefore enhance durability.

Eflornithine hydrochloride 13.9 % cream irreversibly inhibits ornithine decarboxylase, the rate‑limiting enzyme in polyamine synthesis required for keratinocyte proliferation. Twice‑daily application leads to a measurable reduction in hair calibre and growth rate within eight weeks [9]. Although monotherapy is modestly effective, combining eflornithine with laser or IPL has yielded additive benefits in small split‑face studies conducted predominantly in Caucasian cohorts [10,11]. Phototype‑dependent treatment response, however, necessitates validation in darker skin where epidermal melanin competes for incident light and increases the risk of dyschromia [12].

Pakistan ranks among countries with high patient turnover for cosmetic depilation; nevertheless, local evidence on optimal regimens is scarce. Leveraging data extracted from a recently completed randomised controlled trial at Sandeman Provincial Hospital, Quetta, the current article aims to present a full research report comparing IPL combined with topical eflornithine versus IPL alone. Our working hypothesis posited that combination therapy would significantly increase the proportion of women achieving at least a one‑grade improvement on the mFG scale at 24 weeks.

## Materials and methods

This was an open-label, two-arm, parallel-group randomized controlled trial conducted at the Department of Dermatology, Sandeman Provincial Hospital, Quetta, Pakistan, from August 5, 2023, to February 5, 2024. The study protocol received approval from the Research and Ethics Committee of Sandeman Provincial Hospital (CPSP/REU/DER-2021-001-1429) and was conducted in accordance with the Declaration of Helsinki (2013 amendment). The trial was registered with the ISRCTN: The UK's Clinical Study Registry under the identifier ISRCTN47386451. 

Written informed consent was obtained from all participants prior to enrolment. Eligible participants were women aged 18-70 years presenting with visible terminal facial hair, graded as moderate (mFG = 2) or severe (mFG = 3) based on a validated 4-point photographic scale adapted for South Asian skin types [13].

Exclusion criteria comprised pregnancy or lactation; endocrine disorders (polycystic ovary syndrome, late‑onset congenital adrenal hyperplasia, thyroid disease); intake of hormonal therapy, photosensitising medication or anti‑androgens within six months; history of keloids, active facial dermatoses, herpes labialis, light‑triggered seizures; prior adverse reaction to eflornithine or IPL; Fitzpatrick skin type VI; and unwillingness to use broad‑spectrum sunscreen.

Block randomisation (block size 8) with a 1:1 allocation ratio was performed by an independent statistician using a computer‑generated sequence. Allocation concealment employed sequentially numbered opaque sealed envelopes. Because the intervention included a topical medication, participants and treating physicians were not masked; however, outcome assessors and data analysts were blinded to group assignment to mitigate observer bias.

All participants received six IPL sessions at four‑week intervals using a quartz‑filtered xenon lamp platform (Dermalase™ XL, China) fitted with a 640‑nm cut‑off filter. A test‑spot determined initial fluence; typical session‑1 parameters were fluence 18-22 J cm⁻², pulse width 3 ms, triple‑pulse mode with 20‑ms pulse delay, and 10 × 40 mm spot size under continuous contact cooling to 4 °C. Fluence was titrated by 2-4 J cm⁻² per session to a maximum 42 J cm⁻² based on perifollicular edema intensity. The treatment area, bounded cranially by the infra‑orbital rim and caudally by the sub‑mandibular margin, was shaved 24 h pre‑procedure.

Participants randomised to the combination arm additionally applied eflornithine hydrochloride 13.9 % cream (Vaniqa™, Allergan) in a thin layer to treated areas twice daily for 24 weeks. They were instructed to gently remove residual cream before each IPL session and to resume application 12 h post‑procedure. Standardised sunscreen SPF 50 +  was supplied to all participants with directions for liberal application every 3 h during daytime.

The primary outcome was the proportion of women achieving ≥ 1‑grade reduction on the mFG scale at week 24 compared with baseline.

Secondary outcomes were as follows: (i) Mean percentage change in terminal hair count within two pre‑defined 2.5 × 2.5 cm cheek fields was measured with dermascopic digital photography analysed by Image‑J software; (ii) Patient satisfaction was evaluated at week 24 using a standardised 5-point Likert scale (1 = very dissatisfied, 2 = dissatisfied, 3 = neutral, 4 = very satisfied, 5 = extremely satisfied). The questionnaire was administered in person by a blinded study nurse at the final follow-up visit, and participants were instructed to base their responses on perceived hair reduction, cosmetic improvement, and treatment convenience; and (iii) Incidence of treatment‑related adverse events was graded by Common Terminology Criteria for Adverse Events v5.0.

Sample‑size calculation assumed a responder rate of 70 % for IPL alone and 90 % for combination therapy, requiring 69 participants per arm for 80 % power at α = 0.05. Accounting for 10 % attrition, 152 patients were enrolled. Data were analysed per protocol using IBM SPSS Statistics for Windows, Version 26 (Released 2019; IBM Corp., Armonk, New York, United States). Continuous variables are expressed as mean ± SD and were compared using independent‑sample t‑tests or Mann-Whitney U as appropriate. Categorical variables were compared with χ² or Fisher’s exact test, and effect sizes presented as risk differences with 95 % confidence intervals (CI). A two‑sided p‑value < 0.05 denoted statistical significance.

## Results

**Figure 1:**
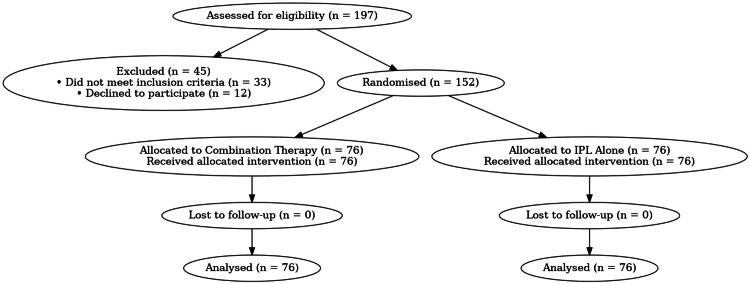
CONSORT diagram. CONSORT: Consolidated Standards of Reporting Trials

A total of 197 women were screened for eligibility, of whom 152 met the inclusion criteria and were randomised. All randomised participants completed the 24-week assessment, resulting in 0 % attrition as shown in Figure 1 (CONSORT diagram). Baseline demographic and clinical characteristics did not differ significantly between groups, as shown in Tables 1, 2.

**Table 1:** Baseline age and duration of hirsutism. IPL: Intense pulsed light

Variable	Combination (n = 76)	IPL Alone (n = 76)
Age, years (mean ± SD)	28.02 ± 10.59	30.76 ± 10.57
Duration, months (mean ± SD)	8.60 ± 3.09	9.21 ± 3.24

**Table 2:** Baseline severity and residence. IPL: Intense pulsed light

Variable	Combination (n = 76)	IPL Alone (n = 76)
Urban/Rural	37/39	31/45
Moderate/Severe	53/23	23/53

Sixty eight of 76 women (89.5 %; 95 % CI 80.4-94.8 %) in the combination arm and 53 of 76 (69.7 %; 95 % CI 58.2-79.0 %) in the IPL‑only arm achieved at least a one‑grade improvement on the mFG scale (risk difference 19.8 %; χ² = 8.7; p = 0.003), as elucidated in Table 3.

**Table 3:** Primary and secondary outcomes at week 24. IPL: Intense pulsed light

Group	Responders (n)	Non-responders (n)	Responder rate % (95% CI)	Mean % hair reduction ± SD	Mean difference (95% CI)
Combination	68	8	89.5 (95% CI 80.4–94.8)	90 ± 12	—
IPL Alone	53	23	69.7 (95% CI 58.2–79.0)	59 ± 18	31% (25.9–36.1)

**Figure 2:**
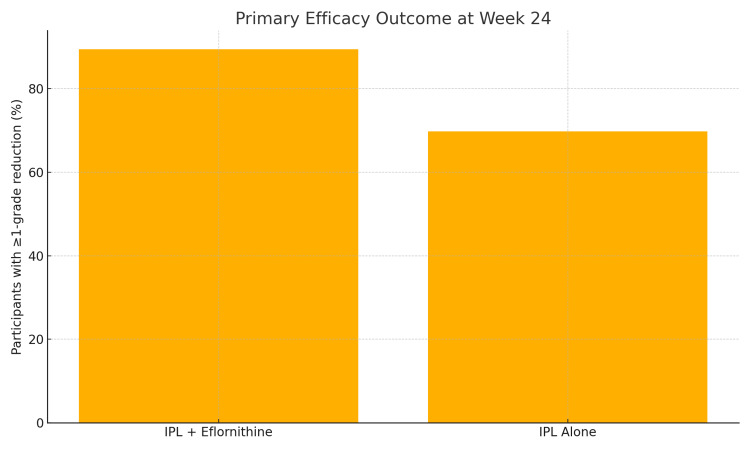
Comparison of responder rates between groups. IPL: Intense pulsed light

The number‑needed‑to‑treat (NNT) to produce one additional responder was five. Mean percentage terminal-hair reduction at week 24 was 90 % ± 12 % with combination therapy versus 59 % ± 18 % for IPL alone (mean difference = 31 %, 95 % CI 25.9-36.1 %; t = 12.1; p < 0.001), as shown in Figure 2.

Patient satisfaction scores mirrored objective findings: 83 % of combination participants rated outcome as ‘very satisfied’ or ‘extremely satisfied’ compared with 57 % in the control group (p = 0.002). Both regimens were well tolerated. The most common adverse event was transient perifollicular erythema (CTCAE grade 1) with a mean duration of 12 ± 4 hours, reported by 32 % and 29 % of participants in the combination and IPL-only groups, respectively (p = 0.68). Mild xerosis (CTCAE grade 1) with a mean duration of 4 ± 1 days occurred in 21 % versus 18 % of participants. No higher-grade events, blistering, post-inflammatory hyperpigmentation, or scarring were observed. No participant discontinued treatment because of adverse events. The superiority of combination therapy was consistent across age strata (< 30 y vs ≥ 30 y), residence (urban vs rural) and baseline severity (moderate vs severe); interaction p‑values were non‑significant. Exploratory linear regression confirmed the therapy group as the sole independent predictor of percentage hair reduction (β = 0.58; p < 0.001).

## Discussion

The present trial demonstrates that adding topical eflornithine to a standard six-session IPL protocol confers a clinically and statistically significant advantage over IPL monotherapy in Pakistani women with idiopathic facial hirsutism. The 20% absolute increase in responder rate aligns with the additive effects observed in smaller bilateral split-face trials from North America and Europe [10,11] and confirms that the mechanistic synergy between pharmacological inhibition of follicular polyamine synthesis and photo-thermal follicular destruction transcends ethnic and phototype boundaries. The additive benefit likely arises from complementary mechanisms: eflornithine slows follicular matrix cell proliferation and prolongs the telogen phase through irreversible inhibition of ornithine decarboxylase, thereby reducing the pool of follicles capable of rapid regrowth. This pharmacological delay synergises with IPL’s photothermal destruction of pigmented follicles during anagen, producing both immediate clearance and extended suppression of regrowth.

Strengths of the study include adequate power, rigorous allocation concealment, blinded outcome assessment and zero attrition, all of which enhance internal validity. Contrary to concerns that eflornithine might increase epidermal photosensitivity, we observed no rise in dyspigmentation or other IPL‑related adverse events, corroborating its favourable safety profile [14]. Importantly, participants appreciated the rapidity of hair‑regrowth delay achievable within the inter‑session interval, reflected in markedly higher satisfaction scores. Our findings must nonetheless be interpreted in the light of certain limitations. First, the open‑label nature could have influenced self‑reported outcomes, although objective digital hair counts corroborated subjective impressions. Second, our follow-up period was limited to 24 weeks, which captures efficacy within the inter-session interval but does not address the long-term durability of hair reduction. Consequently, relapse rates beyond this period and the potential need for booster IPL sessions or continued eflornithine application remain unknown. An observational extension of the present cohort is ongoing to document these outcomes, which will provide valuable data on maintenance strategies and durability of effect. Third, while the selected IPL parameters are representative of commercial practice, comparative trials using long‑pulsed Nd:YAG or diode lasers may be warranted given their deeper penetration in pigmented skin [15,16]. In the present study, Fitzpatrick type VI was excluded due to the higher theoretical risk of post-inflammatory hyperpigmentation and epidermal injury when using IPL with a 640 nm cut-off filter in deeply pigmented skin, where epidermal melanin competes strongly for incident light. While this exclusion enhances participant safety, it does limit generalisability to the darkest skin tones commonly encountered in South Asia. Longer-wavelength platforms such as 1064 nm long-pulsed Nd:YAG lasers, which exhibit reduced melanin absorption and deeper penetration, may be more appropriate for phototypes VI. Future trials incorporating these modalities in combination with eflornithine would be valuable to address this gap. IPL was prioritised here because of its broad wavelength range, capacity to treat large surface areas rapidly, established safety in phototypes II-V when combined with adequate epidermal cooling, and wider availability and lower cost relative to Nd:YAG in the Pakistani public-sector context. These pragmatic considerations support the relevance of our findings for most regional clinical settings, though head-to-head comparisons with Nd:YAG-based regimens remain an important research priority. Finally, the cost‑effectiveness of combination therapy relative to systemic anti‑androgens, waxing or electrolysis remains unexplored in the regional context and should be examined by health‑economic modelling. 

While our trial did not include a formal economic analysis, such studies could assess the cost per additional responder and long-term maintenance costs compared with IPL monotherapy, providing crucial data for decision-making in low-resource South Asian settings where affordability is a primary determinant of treatment uptake.

Future research priorities include extended longitudinal cohorts, head‑to‑head comparisons with emerging topical androgen‑receptor inhibitors such as clascoterone, and exploration of biomolecular predictors of response including single‑nucleotide polymorphisms in the androgen‑receptor and ornithine‑decarboxylase genes [17]. Meanwhile, our data provide robust evidence to support adoption of the IPL‑eflornithine combination as a first‑line, hormone‑sparing option for women seeking definitive yet non‑invasive management of IFH.

## Conclusions

In conclusion, six sessions of intense pulse light combined with twice‑daily topical eflornithine 13.9 % cream yielded significantly superior hair‑reduction efficacy, higher patient satisfaction and comparable tolerability compared with IPL alone in Pakistani women with idiopathic facial hirsutism. With an NNT of five and no incremental safety concerns, the combination represents an evidence‑based, non‑hormonal, culturally acceptable solution for a condition that poses considerable psychosocial burden. Dermatologists and aesthetic practitioners should consider integrating eflornithine into their IPL protocols, particularly for patients seeking rapid, durable results without systemic therapy. These findings warrant confirmation in larger, more diverse cohorts and with extended follow-up to determine whether the combination may offer a durable, cost-effective, non-hormonal first-line option.

## Appendices

Patient satisfaction questionnaire

Dear Participant,

We value your feedback regarding the treatment you have received. Please answer the following questions honestly. Your responses will help us evaluate and improve the quality of care. All answers will be kept confidential.

Instructions: For each question, please circle the number that best represents your level of satisfaction.

Response Scale: 1 = Very Dissatisfied 2 = Dissatisfied 3 = Neutral 4 = Satisfied 5 = Very Satisfied

Questions: 1. How satisfied are you with the reduction in hair density after treatment? ( 1 ) ( 2 ) ( 3 ) ( 4 ) ( 5 ) 2. How satisfied are you with the cosmetic appearance of the treated areas? ( 1 ) ( 2 ) ( 3 ) ( 4 ) ( 5 ) 3. How satisfied are you with the convenience and comfort of the treatment sessions? ( 1 ) ( 2 ) ( 3 ) ( 4 ) ( 5 ) 4. How satisfied are you with the overall impact of the treatment on your confidence and quality of life? ( 1 ) ( 2 ) ( 3 ) ( 4 ) ( 5 ) 5. Would you recommend this treatment to others with similar concerns? ( 1 ) ( 2 ) ( 3 ) ( 4 ) ( 5 )
